# Safety of hydroxychloroquine for treatment or prevention of SARS‐CoV‐2 infection: A rapid systematic review and meta‐analysis of randomized clinical trials

**DOI:** 10.1002/iid3.374

**Published:** 2020-11-26

**Authors:** Alberto Enrico Maraolo, Adriano Grossi

**Affiliations:** ^1^ First Division of Infectious Diseases Cotugno Hospital Naples Italy; ^2^ Section of Hygiene, University Department of Life Sciences and Public Health Università Cattolica del Sacro Cuore Roma Italy

**Keywords:** COVID‐19, hydroxychloroquine, meta‐analysis, randomized controlled trials, safety, SARS‐CoV‐2

## Abstract

**Introduction:**

Hydroxycloroquine (HCQ) has been extensively studied for treatment and prevention of coronavirus diseases 2019 (COVID‐19) from the start of the pandemic. Conflicting evidence about its usefulness has begun to accrue.

**Methods:**

In the face of controversial results about clinical efficacy of HCQ, we performed a rapid systematic review to assess its safety in the framework of COVID‐19 randomized clinical trials.

**Results:**

Five studies investigating 2291 subjects were included. The use of HCQ was associated with higher risk of adverse event compared with placebo or standard of care: odds ratio 4.57, 95% confidence interval 2.14–9.45.

**Conclusion:**

Safety profile of HCQ appears to be unsatisfactory when used to treat or prevent COVID‐19, especially in the light of unproved clinical benefit.

## Graphical abstract

This rapid systematic reviews described that the odds ratio of any adverse event is more than four‐fold higher in patients receiving hydroxychloroquine compared with placebo in the context of treatment or prevention of SARS‐CoV‐2 infection.

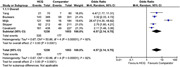

## 1 INTRODUCTION

During the early phases of coronavirus diseases 2019 (COVID‐19) pandemic, the repurposing of drugs used for other indications represented one of the few available options to fight severe acute respiratory syndrome coronavirus 2 (SARS‐CoV‐2), the causative agent of the new clinical entity. Chloroquine (CQ) and its more soluble and less toxic metabolitehydroxychloroquine (HCQ) are two antimalarial drugs derivative from 4‐aminoquinoline employed in rheumatological setting due to their immunomodulatory properties.^1^ In the setting of COVID‐19 pandemic, they have drawn much attention for their potential antiviral activities, combined with other interesting features such as wide availability, inexpensive profile, manageabilityderived from long historical use.^1^

Nevertheless, in spite of promising results from observational studies, evidence synthesis from randomized clinical trials (RCTs) indicates no efficacy of HCQ/CQ on relevant clinical outcomes, except time to symptom resolution, but with low certainty.^2^

In the light of this very modest beneficial effect, we tried to gauge the safety profile of HCQ/CQ in the context of COVID‐19 pandemic on the basis of data from RCTs published so far, to highlight the trade‐off between pros and cons of HCQ/CQ repurposing to treat or prevent SARS‐CoV‐2 infection.

## 2 METHODS

We performed a rapid systematic review and meta‐analysis according to the Preferred Reporting Items for SystematicReviews and Meta‐analyses reporting guidelines (Supporting Information Material S1).^3^ The study protocol was preregistered and made publicly available on osf.io/qckf5.

We searched Medline, Scopus, and Embase for allinvestigations published as peer‐review publicationany time from inception up to August 11, 2020, without language restriction. The details of the search strategy for each database were provided in Supporting Information Material S2.

Inclusion criteria relied on the following PICO strategy: subjects with or without infection by SARS‐CoV‐2 (P—participants), undergoing a course of HCQ/HQ for treatment or prevention, respectively, of SARS‐CoV‐2 infection (I—intervention); comparison according to the study, namely placebo or other comparator such standard of care without antimalarials (C—comparison); safety profile in terms of total adverse events (AEs) as main endpoint (O—outcome). Secondary outcomes werespecific types of AEs (e.g., gastrointestinal, cardiovascular and so on). We performed pooled analyses regarding secondary outcomes if consistent definitions across the RCTs were available in at least two RCTs. Studies were excluded if the intervention (HCQ/HQ) was not administered in an RCT or if the RCT enrolled ≤100 patients to avoid highly implausibleand hugely imprecise effect estimates as described elsewhere.^2^

Information of interest extracted from included studies were: study authors, country, setting (hospitalized or community subjects), time span, type of drug (HCQ or HQ with dosages and potential concurrent agents), type of comparator, indications (prevention or treatment), main characteristics of enrolled patients, sample size, safety populations (the group of individuals receiving at least one dose of intervention or comparator drug and that was evaluated for any safety‐related analysis), number of total AEs in each group and specific AE according to study's definition. Eventually, data regarding the main outcome of each RCT were extracted as well.

The quality of included studies was graded by means of the Rob2 tool (Revised tool for Risk of Bias in randomized trials).^4^ Odds ratios (OR) with 95% confidence intervals (CI) were calculated as summary statistics. The pooled OR was computed with the Mantel‐Haenszelmethod in the framework of a random‐effects model for dichotomous data. The number needed to harm (NNH) was calculated for each study as well, as inverse of the absolute risk increase.

Statistical heterogeneity was measured though *I* ^2^ statistic and was quantified as low, moderate, and high, with upper limits of 25%, 50%, and 75% for *I* ^2^, respectively. To evaluatepotential publication bias, Egger's test was carried out to estimate asymmetry of the funnel plot in case at least 10 studies were retrieved. Sensitivity analyses were performed to assess robustness of findings, in particular resorting to leave‐one‐out method to gauge the impact of each study. A 95% prediction interval was also computed, to define the range of values where observations from future studies will fall.^5^

Results were deemed as statistically significant at *p* < .05. Analyses were performed through Review Manager 5.4 (Cochrane Collaboration).

## 3 RESULTS

A total of five articles,^6^, ^7^, ^8^, ^9^, ^10^ all investigating only HCQ and not CQ, were included in the quantitative analysis from the 417 records screened (the selection process is illustrated in Supporting Information Material S3). The number of examined patients was 2291. Study features are summarized in Table 1. Main outcomes were clinical or virologic efficacy of HCQ and in no case it was superior than comparator.

**Table 1:** Characteristics of the studies included in the meta‐analysis

Author [ref]	Country and type	Setting	Intervention (participants, *n*)	Comparison (participants, *n*)	Baseline features	Primary outcome	Main results	Safety population and most common AE	NNH (any AE), 95% CI
Tang et al.^5^	China, open‐label	Multicenter, adults with mild‐to‐moderate COVID‐19	HCQ + SoC (75) HCQ: 1200 for 3 days as LD, then 800 mg daily for 14 (mild‐to‐moderate forms)–21 days (severe disease)	SoC (75)	Mean age 46.1 years, male sex 55%, mild disease 15%, moderate disease 84%	Negative conversion of SARS‐CoV‐2 by 28 days	Probability of positive outcome 85.4% vs. 81.3% (difference equal to 4.1%, 95% CI: 10.3%–18.5%)	70 vs. 80—nausea (10%) vs. several AEs (1%)	4.7, 3.0–10.8
Boulware et al.^6^	Canada and United States, double‐blind	Multicenter, adults who had high‐risk exposure to people with confirmed COVID‐19 (within 4 days)	HCQ (414) HCQ: 800 mg as LD, followed by 600 mg in 6‐8 h, then 600 mg daily for 4 additional days (5 days total)	Placebo (407)	Median age 41 vs. 40 years, male sex 47.3% vs. 49.4%, no comorbidities 73.9% vs. 71.3%	Incidence of either laboratory‐confirmed Covid‐19 or illness compatible with COVID‐19 within 14 days	Incidence 11.8% vs. 14.3% (absolute difference was −2.4%, 95% CI: 7.0–2.2)	349 vs. 351 –diarrhoea, abdominal discomfort, or vomiting (23.2%) vs. nausea or upset stomach (7.7%)	4.3, 3.4–5.9
Mitjà et al.^7^	Spain, open‐label	Multicenter, nonhospitalized adult patients with confirmed COVID‐19	HCQ (136) HCQ: 800 mg as LD, followed by 400 mg daily for 6 additional days (7 days total)	Usual care (157)	Mean age 41.6 vs. 41.7 years, male sex 27.9% vs. 34.4%, no comorbidities 47.8% vs. 45.9%	Reduction of viral RNA load in nasopharyngeal swabs at Days 3 and 7 after treatment start	No significant differences about the mean reduction of viral load at Days 3 and 7	169 vs. 184—gastrointestinal disorder 88.1% vs. infections/infestations 6.6%	1.6, 1.4–1.8
Skipper et al.^8^	Canada and United States, double‐blind	Multicenter, nonhospitalized adults with probable or laboratory‐confirmed COVID‐19	HCQ (212) HCQ: 800 mg once as LD, followed by 600 mg in 6‐8 h, then 600 mg daily for 4 more days	Placebo (211)	Median age 41 vs. 39 years, male sex 42% vs. 45.5%. no comorbidities 66.0% vs. 69.7%, confirmed case 34.4% vs. 34.1%	Change in global symptom severity over 14 days as longitudinally measured on a 10‐ point visual analogue scale	No significant differences in symptom severity (absolute −0.27 points, 95% CI: 0.610.07 points]	212 vs. 211—nausea or upset stomach (31.1% vs. 12.3%)	4.6, 3.3–7.8
Cavalcanti et al.^9^	Brazil, open‐label	Multicenter, hospitalized adults with probable or laboratory‐confirmed COVID‐19	HCQ +/− azithromycin (438) HCQ: 400 mg twice a day for 7 days	Usual care, neither HCQ nor azithromycin or azithromycin without HCQ (227)	Mean age 51.3 years HCQ alone (49.6 plus azithromycin) vs. 49.9, male sex 60.5% vs. 54.2%, confirmed cases 75.6% vs. 76.2%	Clinical status at 15 days as assessed with the use of a 7‐level ordinal scale	Odds of having a higher score on the 7‐point ordinal scale at 15 days were not affected by either hydroxychloroquine alone or hydroxychloroquine plus azithromycin	438–227—low lymphocyte level (10.5%) vs. thrombocytopenia (8.4%)	6.0, 4.2–10.8

Abbreviations: AE, adverse event; CI, confidence interval; COVID‐19, coronavirus diseases 2019;HCQ, hydroxychloroquine; LD, loading dose; NNH, number needed to harm; SoC, standard of care; vs, versus.

**Figure 1:**
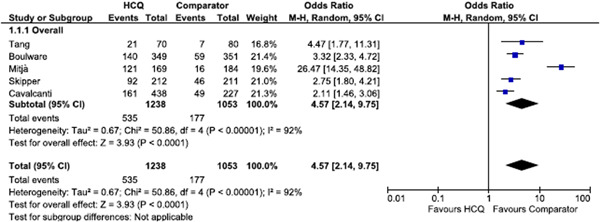
Odds ratio of adverse events between subjects receiving HCQ and the ones receiving the comparator. HCQ, hydroxycloroquine

Overall, the proportion of AE was 43.2% (535/1238) in the HCQ group versus 16.8% (177/1053) in the comparator group. The use of HCQ was associated with higher risk of AE compared with placebo or standard of care: OR: 4.57, 95% CI: 2.14–9.45, with relevant heterogeneity (*I* ^2^ = 92%), as described in Figure 1. Funnel plot was not drawn due to the low number of included studies. The type of most common AE varied but the ones involving the gastrointestinal tract were the most frequent. When singled out specific AE, risk was always higher among subject receiving HCQ, but statistical significance was met only for nausea and skin reactions (Table 2). In detail, OR for experience nausea was 3.31 (95% CI: 2.38–4.61), skin reaction was 3.71 (95% CI: 1.11–12.43). Concerning serious AEs, OR was 1.11 (95% CI: 0.45–2.79). NNH was quite low, ranging from 1.6 to 6.

**Table 2:** Pooled analyses concerning the risk of specific adverse events (subjects receiving hydroxychloroquine versus placebo)

Safety outcome	Included studies, *n*	Total sample, *n*	Event/total (HCQ group)	Event/total (comparator)	OR, 95% CI	*p* Value	*I* ^2^ statistics
Arrythmia	4	1591	7/889	1/702	3.24, 0.55–19.04	.194	0%
Change in vision	4	1626	13/800	5/826	2.43, 0.60–9.87	.214	27.5%
Ear labyrinth disorder	4	1626	21/800	9/826	2.08, 0.93–4.64	.074	0%
Headache	2	1123	15/561	4/746	1.01, 0.34–3.55	.877	17.1%
Increased liver enzymes	2	810	27/508	9/307	1.69, 0.78–3.68	.185	0%
Nausea	4	1938	162/1069	55/869	3.31, 2.38–4.61	<.001	0%
Neurological manifestations such as dizziness, vertigo, irritability	3	1476	102/730	29/746	4.10, 0.78–21.47	.095	91.3%
Severe adverse event	3	1168	17/677	14/491	1.11, 0.45–2.79	.817	15.7%
Skin reactions	3	1476	21/730	4/746	3.71, 1.11–12.43	.034	16.61%
Warmth, hot flashes, night sweats	2	1123	2/561	1/562	1.36, 0.10–19.33	.820	30.8%

Abbreviations: CI, confidence interval; HCQ, hydroxycloroquine.

Quality assessment was described in Supporting Information Material S4: the risk of bias was low in most studies. Of note, three of them were open‐label,^6^, ^8^, ^10^ being the other two double‐blind.^7^, ^9^ Sensitivity analysis confirmed the robustness of the main finding: the omission of whichever study, including the largest one^7^ or the one with the greatest OR,^8^ changed neither the direction nor the strength of the association between use of HCQ and risk of AE (Supporting Information Material S5). Last, the 95% prediction interval ranged from 0.26 to 80.46.

## 4 DISCUSSION

This is the first systematic review focusing on safety profile of HCQ/CQ in the context of COVID‐19 summarizing data only from high‐quality RCTs. The main finding was the higher risk of AE (more than fourfold) in individuals receiving HCQ compared with placebo or standard of care. Although the magnitude of the effect across the studies varied, the direction of effect in all RCTs pointed to a disadvantageous safety profile of the 4‐aminoquinoline derivative unambiguously. Gastrointestinal AEs were the most frequently reported but among the most clinically relevant change in vision, arrythmia, and neurological manifestation stood out. The biological plausibility of these events is underpinned by sound data from both historical cohorts and worldwide pharmacovigilance.^11^

Results are coherent with a previous evidence synthesis collecting data only from RCTs, but not limited to COVID‐19, being focused on other settings such as rheumatological diseases characterized by different HCQ schedules and including fewer studies in the framework of treatment or prevention of SARS‐CoV‐2 infection.^12^

Actually, the hypothesis that HCQ couldalter viral growth of SARS‐CoV‐2 was based on weak evidence and inconsistent results from other viral infections in vitro, in animals, and in clinical studies.^13^ Additionally, the likelihood of benefit, on the basis of poor mechanistic reasoning, is quite limited, also taking into account the high doses used so far in COVID‐19, increasing the risk of AEs and of hazardous interactions with commonly used drugs such as macrolide antibiotics.^14^ Indeed, a very recent meta‐analysis of both RCTs and observational studies demonstrated that HCQ alone was not associated with reduced death risk in hospitalized COVID‐19 patients but its combination with azithromycin significantly increased mortality (relative risk 1.2, 95% CI: 1.04–1.54).^13^

The hype generated by very first observational studies on HCQ was quite soon contested by the emergence of serious flaws in design and analysis of these works, calling for high‐quality RCTs to properly assess the clinical usefulness of the drug.^15^

The RCTs included in this meta‐analysis included nonsevere COVID‐19 patients (or healthy subjects who were at risk of being close contact of confirmed cases), thus it is reasonable to affirm that the risk of AEs could be even amplified in more fragile individuals, the ones with serious forms of disease.

Notwithstanding, some limitations of the present study need to be underlined. For starters, studies investigating CQ were not retrieved, therefore, all results and findings are related to HCQ only. Then, populations were heterogeneous: COVID‐19 patients (although the most suffering from mild or moderate disease) and subjects at risk of SARS‐CoV‐2 infection, having different baseline likelihood to experience AE. Heterogeneous were doses and durations of HCQ as well. Comparators were different and not rarely HCQ was used in combination with other agents such as azithromycin in one study,^10^ and, in that case, it was impossible to disentangle the effects of HCQ from the one of companion drugs. Furthermore, not all information could be pooled due to inconsistent definition of AE, limiting the number of feasible analyses and so potentially explaining why most results did not reach statistically significance. Moreover, the profile of AEs was very broad, also including minor occurrences usually not interfering with medicine adherence, drug efficacy, daily activities and not seriously threatening patients' health. Open‐label studies were potentially prone to ascertainment bias at least with regard to subjective AEs. Eventually, the prediction interval was very wide, even including values conferring a protective role of HCQ toward AEs versus comparator and so lowering the confidence in summary estimates, but this can mirror the clinical heterogeneity of the studies.

Nevertheless, this kind of heterogeneity, regarding patients' features and protocols, involve neither methodological nor statistical aspects: all works were RCTs and magnitude as well as direction of the effect were consistent across studies.

In conclusion, given the limited and controversial, if any, benefit linked with HCQ when used to treat or prevent SARS‐CoV‐2 infection, the burden of AE overwhelms the potential advantages. HCQ should be used solely in the framework of clinical trials for COVID‐19. Routine use in clinical practice currently should be avoided on the basis of an unfavourable trade‐off between benefits and harms.

## CONFLICT OF INTERESTS

The authors declare that there are no conflict of interests.

## AUTHOR CONTRIBUTIONS

All authors contributed to the study conception and design. Literature search and data extraction were performed by both the authors. Alberto Enrico Maraolo carried out the data analysis and drafted the manuscript, critically revised by Adriano Grossi.

## Supporting information

Supporting information.

Supporting information.

Supporting information.

Supporting information.

## Data Availability

The data that support the findings are publicly available, being freely retrievable from the open‐access published studies that were meta‐analysed, in particular from: Tang et al. https://www.bmj.com/content/369/bmj.m1849; (https://doi.org/10.1136/bmj.m1849); Boulware et al. https://www.nejm.org/doi/full/10.1056/NEJMoa2016638 (https://doi.org/10.1056/NEJMoa2016638); Mitjà et al. https://academic.oup.com/cid/advance-article/doi/10.1093/cid/ciaa1009/5872589 (https://doi.org/10.1093/cid/ciaa1009); Skipper et al. https://www.acpjournals.org/doi/10.7326/M20-4207 (https://doi.org/10.7326/M20-4207); Cavalcanti et al. https://www.nejm.org/doi/full/10.1056/nejmoa2019014 (https://doi.org/10.1056/NEJMoa2019014).
