# Supplementary material for: Safety of hydroxychloroquine for treatment or prevention of SARS‐CoV‐2 infection: A rapid systematic review and meta‐analysis of randomized clinical trials
Source: Immun Inflamm Dis. 2020 Nov 26;9(1):31–6. doi: 10.1002/iid3.374 (PMC7753686; doi:10.1002/iid3.374)
Supplement: Supplementary file 2 — Supporting information. [file IID3-9-31-s002.docx]

**Supplementary Material 2:** Literature search strategy

| **MEDLINE** | (covid-19[Title/Abstract] OR "SARS-CoV-2"[Title/Abstract] OR coronavirus[Title/Abstract]) AND (chloroquine[Title/Abstract] OR hydroxychloroquine[Title/Abstract]) AND (randomized[Title/Abstract] OR randomised[Title/Abstract]) |
| --- | --- |
| **EMBASE** | ('covid 19':ab,ti OR 'sars-cov-2':ab,ti OR coronavirus:ab,ti) AND (chloroquine:ab,ti OR hydroxychloroquine:ab,ti) AND (randomized:ab,ti OR randomised:ab,ti) |
| **SCOPUS** | (covid-19 OR "SARS-CoV-2" OR coronavirus) AND (chloroquine OR hydroxychloroquine) AND (randomized OR randomised) |
