# Supplementary material for: Safety of hydroxychloroquine for treatment or prevention of SARS‐CoV‐2 infection: A rapid systematic review and meta‐analysis of randomized clinical trials
Source: Immun Inflamm Dis. 2020 Nov 26;9(1):31–6. doi: 10.1002/iid3.374 (PMC7753686; doi:10.1002/iid3.374)
Supplement: Supplementary file 3 — Supporting information. [file IID3-9-31-s003.docx]

**Supplementary Material 3:** Results of literature search and flow diagram for selection of eligible studies.

Studies included in full-text screening
(n = 6)

Records screened
(n = 224)

Records identified through databases searching

from inception to 11/08/2020 (n = 417)

- Medline 117
- Embase 111
- Scopus 189

## Identification

Records after duplicates (n = 193) removed

(n = 224)

## Screening

Records excluded
(n =218)

Studies excluded upon full-text review, with reasons (n = 1):

- Study enrolling ≤ 100 patients (1*).

## Eligibility

## Included

Studies included in quantitative analysis

(n = 5)

* Reference of the excluded study: Chen J, Liu D, Liu L, Liu P, Xu Q, Xia L, et al. A pilot study of hydroxychloroquine in treatment of patients with moderate COVID-19. Zhejiang da xue bao Yi xue ban = Journal of Zhejiang University Medical sciences 2020; 49: 215-219. doi: 10.3785/j.issn.1008-9292.2020.03.03
