# Supplementary material for: Safety of hydroxychloroquine for treatment or prevention of SARS‐CoV‐2 infection: A rapid systematic review and meta‐analysis of randomized clinical trials
Source: Immun Inflamm Dis. 2020 Nov 26;9(1):31–6. doi: 10.1002/iid3.374 (PMC7753686; doi:10.1002/iid3.374)
Supplement: Supplementary file 4 — Supporting information. [file IID3-9-31-s004.docx]

**Supplementary Material 5:** Sensitivity analysis according to the leave-one-out method.

| Study omitted | Odds ratio | 95% Confidence interval |
| --- | --- | --- |
| Tang | 4.61 | 1.94-10.95 |
| Boulware | 5.04 | 1.72-14.82 |
| Mitjà | 2.79 | 2.15-3.61 |
| Skipper | 5.27 | 1.94-14.35 |
| Cavalcanti | 5.66 | 2.19-14.63 |
